# Advancement of life and death education research: recommending implementation of the Life + Death Education Framework for teaching and research purposes

**DOI:** 10.3389/fpubh.2024.1440750

**Published:** 2024-11-26

**Authors:** Huy P. Phan, Bing Hiong Ngu, Chao-Sheng Hsu, Si-Chi Chen

**Affiliations:** ^1^School of Education, University of New England, Armidale, NSW, Australia; ^2^College of Education, National Taipei University of Education, Taipei, Taiwan

**Keywords:** Life + Death Education Framework, life care, life enhancement, self well-being, positive psychological personal, life education, death education

## Abstract

*Life* and *death education* is a distinct field of study that has potential practicality and life relevance for us to consider. For example, one notable inquiry pertaining to life education teaching entails appreciation and theoretical understanding of quality life functioning (e.g., a person’s desire to attain spiritual wisdom vs. a person’s desire to attain immense financial wealth). Our research undertakings recently involved the development of a blueprint or framework, which we termed as the ‘Life + Death Education Framework’. This framework is intended to provide relevant information that may serve to assist educators, stakeholders, caregivers, etc. with their teaching practices of life and death education. We premise that to date, there is no clear consensus or agreement among educators as to what one is expected (e.g., specific learning outcome) to teach to students who wish to study and learn about life and death education (e.g., do we introduce to students the metaphysical lens about death?). Moreover, from our point of view, the Life + Death Education Framework may yield insightful guidelines and life-related benefits, such as the heightening of a person’s well-being and/or his or her daily life functioning. As such, then, the focus of the present theoretical-conceptual article is for us to provide an in-depth narrative of the Life + Death Education Framework and how this framework, or potential universal blueprint, could help introduce and clarify our proposition of a life functioning-related concept known as ‘self well-being’. Self well-being, for us, is an alternative nomenclature that may be used in place of subjective well-being.

## 1 Introduction

*Life* (1–3) and *death* (4–6) *education* is an interesting subject discipline in the social sciences for teaching and research development. Broadly speaking, as an introduction, life and death education teaching explores the intimate relationship between life and death. More recently, via means of philosophical reasoning, we have added an additional element – namely, one’s trans-mystical understanding of life and death (7), which in this case includes the premise of ‘post-death experience’ or what we term as ‘the thereafter’ (7, 8). For example, is it plausible that life, death, and the afterlife exist on a continuum?

We premise that the theoretical tenets of life and death education (3, 6, 9, 10), commonly referenced as one distinct subject (as opposed to ‘life education’ and ‘death education’), may coincide with the study of *human agency* (11–13) and the study of *personal well-being* (14–16). That human agency (e.g., a student’s state of determination to seek his or her own career pathway), in its totality, may detail and/or include the mentioning of a person’s ‘quality life functioning’ on a daily basis. In a similar vein, life education teaching (1–3) may help to explain and/or support the intricate nature of one’s personal well-being experience (e.g., how can life education learning help improve one’s personal well-being?).

The present theoretical-conceptual article introduces a philosophical narrative that may coincide with and support the scope of *Frontiers in Public Health* – namely: the potential use of life and death education to help understand the underlying nature of one’s well-being functioning on a daily basis. This line of inquiry, importantly, enables us to introduce a blueprint, or a framework, that we term as the ‘Life + Death Education Framework’. We contend that the Life + Death Education Framework, which we overview and discuss in this article, has immense theoretical and practical potentials for educators, researchers, stakeholders, etc. to consider. Central to this thesis is the extent to which relevant aspects detailed in the Life + Death Education Framework (e.g., essential themes of life and death education for teaching and learning) could help explain the underlying nature of the intricacies (e.g., improvement) of personal well-being (14–16). For example, how can an educator use his or her theoretical understanding of the Life + Death Education Framework to teach students the concept of personal well-being?

Overall, then, the focus of our theoretical inquiry is to use the Life + Death Education Framework to advance the study of personal well-being (14–16). Specifically, our research advancement involves the use of the Life + Death Education Framework to propose an alternative nomenclature and/or concept, which we term as ‘self well-being’ (i.e., in place of ‘personal well-being’). That the underlying nature of self well-being, in this case, consists of and/or incorporates different facets of life and death education teaching. We begin our theoretical-conceptual article by overviewing the premise of life and death education teaching, followed by our concise explanatory account of the Life + Death Education Framework and a detailed overview of our proposition of the concept ‘self well-being’.

## 2 The importance of life education: a brief overview

To our knowledge, we are the first researchers or one of the first researchers to situate the concept of personal well-being (14–16) within the study and/or the context of life education (3, 10, 17, 18). This considered viewpoint, we contend, is novel and innovative given that life education teaching imparts relevant insights that may serve to advance the study of the nature of personal well-being. *Life education*, in terms of focus, considers the overarching emphasis of a person’s life functioning (2, 3). “Why do I want to live?,” “What is the meaning of life and/or in life?,” and “What do I want to do in life?” are sample questions that emphasize the nature of proactive life functioning.

We recently published a theoretical-conceptual article, where we detailed the basic philosophy of the nature of life education teaching (8). We contend that the subject context of life education makes its teaching (3, 10, 17, 18), at times, somewhat philosophical and transpersonal. Life education teaching, ‘soft pure theoretical’ and ‘soft applied’ in terms of intellectual classification (19, 20), differs from other subject disciplines, such as Educational Psychology, Mathematics, Physics, and the like. As an introductory account, the uniqueness of life education, in this case, relates to the teaching and learning of the following tenets:

- Appreciating the fact that life education teaching, which differs from death education (4, 6, 9), is proactive and positive. For example, life education emphasizes the importance of the functioning of *positive attributes* such as aspiration (21), celebration (22), hope (23), optimism (24), and the like. A student’s aspiration to enter medical school, in this analysis, may serve to motivate him or her to strive for exceptional success.
- Recognizing the proactivity of life functioning, which coincides with the study of *human agency* or *agentic engagement* (25, 26). Motivation, aspiration, optimism, and similar attributes are proactive and may serve to energize a person’s state of functioning in life (e.g., a student’s state of motivation to seek mastery of Algebra). Proactivity, contrasting to the attributes of pessimism (27), procrastination (28), disengagement (29), etc., showcases and/or reflects a firm belief in achievement of optimal best and personal experience of flow.
- Emphasis pertaining to the notion of what is known as ‘life trajectories’ or ‘life courses’ (7, 30). The term life trajectory, in this case, suggests that every single one of us has a broad course in life that we strive to attain. A person’s lifespan, in this analysis, may consist of him or her having multiple life trajectories (e.g., a life trajectory of him being a part-time student *and* a life trajectory of him being a full-time bank employee). This life education teaching contends that a person’s ‘positive life experience’ may consist of different perceptions of successful life attainment – for example, a person’s satisfaction, which arises from his or her attainment of financial wealth (i.e., materialistic attainment) vs. a person’s self-fulfilment, which arises from his or her self-transcendence experience (i.e., spiritual, non-materialistic attainment).
- Understand and appreciate the importance or significance of ‘life contexts’ – that perceived multiple life contexts and subsequent experiences (e.g., the life context/experience of one experiencing temporary financial constraint vs. the life context/experience of one experiencing a successful promotion at work) give rise to the formation of what is known as a ‘holistic self’ (8), which details a person’s ‘multiple selves’. In this analysis, at any moment in time and/or in context, a person may manifest or exhibit multiple selves – for example, a teenager’s self of her as being a secondary school student (i.e., ‘Self 1’) vs. her perceived self as a sibling to her brother and sisters (i.e., ‘Self 2’) vs. her perceived ‘self’ as a daughter to her parents (i.e., ‘Self 3’).
- Appreciating the non-definitive, non-conclusive premise of life education teaching. What does the notion of the ‘essence’ of life functioning actually entail? This teaching focuses on the importance and applicability of diversity in viewpoints and interpretations, reflecting different beliefs, values, perceptions, etc. about life functioning. For example, anthropological grounding and historical-sociocultural upbringing (e.g., an Indonesian child who is reared and grows up in South Africa) may serve to instill the belief in the self-fulfilment and gratification of ‘spiritual wisdom’ (31–33) as opposed to, say, the belief in the successful attainment of formal qualifications. Non-financial and/or non-materialistic attainments, in this analysis, showcase differing understandings of positive life qualities, such as the personal experience and gratification of spirituality (e.g., sharing knowledge of Buddhist spirituality with others in the community).
- The teaching of the acquirement of what is known as ‘life wisdom’ (33–35), and the ‘transformation’ of this acquired life knowledge for daily life purposes (36). This premise encapsulates the importance of *applied practice* or the nexus between life education knowledge and practicality. For example, non-academically, a Buddhist monk may utilize his personal understanding of Buddhist spirituality (31, 33, 37) to assist others in the community. In a similar vein, a university student may use her understanding of Maslow’s (38) humanistic teaching to embrace and appreciate the significance of self-transcendence experiences.

### 2.1 Life: pursuing life ideals and human perfection

Our research inquiries and teaching experiences of life education (3, 10, 17, 18) have led us to surmise that personal understanding of life functioning is open-ended with different transpersonal interpretations (e.g., what is the purpose(s) of life? may yield different interpretations). For us, personally, the teaching of life education emphasizes one notable thesis: the ‘cultivation’ of quality life functioning (i.e., the notion of ‘life cultivation’). Cultivation of life functioning, or the notion of ‘life cultivation’ (3, 30), reflects and showcases the practice of growth and positivity, unlike deficit reasoning, which seeks to focus on remedy and preventive measures. How can I flourish?, What can I do to improve my health functioning?, and How can I enjoy life? are typical questions and/or phrases that place emphasis on the practice of cultivation (31, 39, 40).

Cultivation (31, 39, 40) is a proactive process, which seeks to promote and foster life wisdom and different types of characters, values, experiences, etc. for quality life functioning. Existing writings from a number of scholars including us, for example, have delved into the notion of what is known as ‘spiritual cultivation’ (3, 30, 31, 41), or the cultivation of spiritual faith or spiritual belief (e.g., the cultivation of ‘Buddhist spirituality’). One typical practice or engagement that may assist with the process of cultivation, in this analysis, involves the use of meditation (42–44) and personal understanding and perceived feeling of mindfulness (45–47). Our philosophical teaching to students contends that proactive meditation practice could, in fact, assist to cultivate individuals to experience perceived feelings of contentment, inner fulfilment, peace, calmness, and other similar attributes.

The underlying premise then, from the preceding sections, is that life education teaching (3, 10, 17, 18) focuses on or places emphasis on the *pursuing of life ideals* and *human perfection*. This tenet or indication is philosophical at best and may simply reflect a utopian discourse that is unattainable. ‘Life ideals’ are altruistic and/or moralistic endeavors that all of us may aspire to. For example, a teenager learning about life education may aspire to and/or wish to pursue the life ideals of having a pacifist society where there is love, a unifying world where there is no conflict, a community that practices love, peace, and harmony, etc. In a similar vein, the notion of ‘human perfection’ is an interesting but unattainable endeavor for many of us or all of us to accomplish. Individuals and societies are not perfect. Broadly speaking, at a societal level, we are confronted with difficulties, obstacles, conflicts, problems, etc. on a daily basis. At an individual level, likewise, a person may recognize that he or she has numerous deficiencies or shortcomings to overcome that require some form of remedy, resolution, prevention, etc. (e.g., a student acknowledges that he is impatient, or a father’s own omission that he has an anger management issue).

Pursuing life ideals and human perfection, we contend, are utopic, unrealistic, and/or unattainable. *Striving* to attain life ideals and human perfection, however, is euphoric, inspirational, life-changing and, of course, self-fulfilling. It is, in fact, the embodiment of the ‘positivity’ of life education teaching. What does this reference actually mean? Basically, as the term connotes, life education teaching (3, 10, 17, 18) is intended and/or is structured to impart philosophical insights and humanistic understandings into the positive nature of life functioning. It is, for us, a subject area that is esthetic, altruistic, and inspirational. Its intent, in this analysis, is to introduce to society and individuals the following:

- An appreciation for the personal belief in human perfection and the attainment of life ideals.
- An appreciation for the esthetic and altruistic nature of life functioning, showcasing reverence or respect for different life trajectories.
- Seeking to cultivate or to nurture the different types of life qualities for successful life adaptation and life purposes.
- Concerted attempts to contemplate and seek meanings to the true purpose(s) of life and in life (e.g., seeking to experience an altruistic state).
- Concerted attempts to be happy and to live a cherished and self-fulfilling life.

## 3 Death education: an overview

*Death education* (4, 6, 48) is somewhat different from life education and seeks to understand the personal experience of death and dying. For us, life is intimately linked to death. In other words, life education teaching (3, 10, 17, 18) closely associates with death education teaching. In order to understand the true nature of death and death-related matters (e.g., “How can I overcome grief?”), one has to understand the nature of life. We acknowledge that unlike life education, which is positive, esthetic, and aspirational, the subject of death and dying is grim, dark, and somewhat depressing for any person to study, learn, research, etc. Having said this, however, we note that death education teaching also imparts interesting and valuable aspects – for example: the philosophical understanding of death (e.g., does death also mean the demise of one’s state of consciousness?), and comparative cultural beliefs and values pertaining to death (7, 48, 49).

Death education, in brief, relates to the formal teaching and learning of death and death-related matters (4, 5, 36). Death in its simplistic form relates to the demise or the ceasing of life (30). Death education teaching can, in fact, be both pragmatic and philosophical. The notion of pragmaticism of death, in this analysis, entails actual, real-life contexts for consideration, acknowledgement, practice, understanding, etc. For example, Bollig and his colleagues [e.g., (50–52)] have written about the importance of palliative care (53, 54), given that increasingly, many choose to die at home. What educational discourse is available, in person and/or online, that may help family members? Do current initiatives pertaining to ‘Public Palliative Care Education’ (50, 52), also known as ‘PPCE’, require any refinement and/or alternative(s)? These sample questions pertaining to understanding and practice of palliative care, we contend, emphasize the pragmatic nature of death education.

In a similar vein, pragmatic teaching of death education also seeks to understand the complexity and process of grief, loss, and bereavement (55–57). What does a person go through as he or she encounters the loss of a loved one? Is there anything that can be done to help family members overcome their grief? What formal education is available to inform us and/or to impart understanding of grief, loss, and bereavement? This teaching focuses on coping mechanisms (e.g., the use of spiritual faith as a coping mechanism) that may help alleviate the negative emotions and/or feeling that often associate with grief and bereavement.^1^ In a similar vein, death education teaching by the late Elisabeth Kübler-Ross details five stages of grief: *denial* (e.g., “This can’t be me; I am perfectly fine”), *anger* (e.g., “Surely not! Why me, of all people?”), *bargaining* (e.g., “Please, let me live a few more years ….”), *depression* (e.g., “I’m so depressed at the moment; what’s the point? I’m going to die anyway….”), and *acceptance* (e.g., “It’s going to be okay….”).^2^

Death education (4, 5, 36), as we mentioned, is not something that many would choose to study and learn. One only has to read about war ravaged countries, natural disasters, poverty-related deaths, and other life-related negativities to recognize the profound impact of death and dying. By all accounts, death education is morbid and non-esthetic for teaching and learning. Our scholarly research work in the area of holistic and positive life functioning [e.g., (8, 58–60)], however, has led us to consider an alternative position: that it is still possible to view and approach death education teaching, despite its morbid and dark nature, with a sense of positivity (i.e., the perception of death education as being esthetic).

### 3.1 Philosophical understanding of death education

Philosophical teaching of death, which we engage in with our undergraduate and postgraduate students, is non-pragmatic and encourages introspection and personal contemplation for appreciation and meaningful understanding purposes. We contend that death education teaching (4, 5, 36), in this analysis, may embrace the use of a transpersonal or trans-mystical lens (7). A trans-mystical position, in this case, encourages and/or focuses on the following:

- Stimulating intellectual curiosity for the seeking of knowledge, which delves into the ‘unknowns’ of life and death. Known unknowns and unknown unknowns of life (e.g., is there is scientific logic to the study of premonition?) and death (e.g., where does one’s state of consciousness and/or one’s soul go after death?) are somewhat ‘mystical’ (61–63) or trans-mystical (7), and may situate outside the realm of realistic objectivity and/or the realm of ordinary human psyche. Seeking to understand and to appreciate what we do not know about the broad universal contexts at large (e.g., the logic of what is known as ‘post-death experience’) may, in fact, help to alleviate our negative emotions and feelings (e.g., grief) pertaining to death. For example, deep, meaningful understanding of the tenet of saṃsāra (64, 65) or the concept of ‘reincarnation’ (66–68) may assist a person to appreciate and/or to welcome death at any moment in time.
- Concerted attempts to study, learn, and appreciate the importance of diversity in cultural practice, viewpoint, interpretation, perspective, etc. of death. This line of teaching inquiry of death education, as reflected by numerous writings from scholars in China (5), Malaysia (4), Taiwan (9), Tibet (69), Tonga (70), and Vietnam (48), suggests that historical-sociocultural contexts play a notable role. For example, aside from paying respect and/or reverence for the dead, some cultural groups (e.g., Chinese families) firmly believe that partaking in the cultural ritual of ‘ancestor worshipping’ (71–73) may also enable them to communicate with loved ones who have moved one (i.e., that the practice of ancestor worshipping serves as a form of ‘spirit communication’). In a similar vein, many Taiwanese in Taiwan believe in what is known as ‘the Underworld tour’ or ‘Guan Luo Yin’, a ritual that may enable one to communicate with the dead (30).

The mentionings above are theoretical examples that we use to support our philosophical teaching of death education. Central to this teaching practice is the emphasis, which seeks to introduce to students the importance of trans-mystical understanding of the unknowns and ‘abnorms’ of death – for example, is there anything beyond death (7, 8)?, and what does the ritual of ‘ancestor worshipping’ (71, 72, 74) actually connote? This theoretical approach (e.g., a focus on the transpersonal nature of death), somewhat metaphysical in nature, differs from the pragmatic approach (52, 55, 56) and imparts and/or encourages non-definitive and inconclusive issues for us to contemplate.

## 4 Diversity and the issues of universality and generality

There are some academic topics and subjects that are objective and relatively straightforward, showcasing clearness and consistency in terms of interpretation, viewpoint, understanding, etc. For example, the topic of Algebraic Equations such as the solving of simplification of ‘2(*x* + 4) + 3(*x* – 5) – 2*y* = 0’^3^ always yields comparable or consistent understanding, regardless of where it is taught (e.g., a teacher teaching 2(*x* + 4) + 3(*x* – 5) – 2*y* = 0 in South Korea and a teacher teaching 2(*x* + 4) + 3(*x* – 5) – 2*y* = 0 in Australia). In other words, from our viewpoint, there is some form of ‘universality’ or ‘generality’. Universality, in this case, espouses standardization, consistency, and proper structure, regardless of learning and/or sociocultural contexts.

Our research and teaching experiences inform us that universality or generality does not necessarily apply to the case of life and death education (3, 6, 9, 10). Our aforementioned narrative has included some examples (e.g., personal understanding of a need to experience self-transcendence), which may serve to highlight the ‘non-universal’ nature of life and death education teaching. There is, for example, a clear difference in opinion, idea, and/or interpretation with regard to our mentioning of the ‘continuum of life, death, and the thereafter’ (8). In this analysis, we contend that not everyone and/or every culture is inclined to accept and/or to embrace the tenet of trans-mystical or metaphysical experiences. As such, then, some aspects or many aspects in life and death education are somewhat non-concrete and non-definitive, giving rise to a wide range of viewpoints.

One interesting facet that we note is the impact of the contextual environment or the ‘contextual milieu’ at large. This mentioning of the contextual milieu, in part, associates with and reflects the theoretical premise of ‘situated cognition’ (75, 76) and Uri Bronfenbrenner’s (77, 78) *bioecological systems theory*. In this analysis, a person’s development (e.g., his or her cognitive growth) is ‘embedded’ or is situated in context. For example, a child’s cognitive growth is likely to flourish somewhat differently if his or her society places strong emphasis on technological advances for usage (e.g., the child, in this case, is likely to grow up, wishing to be a software engineer). A society or a culture that is technologically deprived, in contrast, is more likely to constrain a child’s cognitive growth. Limited resources (e.g., the provision of high-speed internet connection), in this analysis, may negate opportunities for technological appreciation. As a result, then, anthropological grounding and historical-sociocultural context and upbringing (e.g., a German child who is reared and grows up in Mongolia) may shape a person’s interpretation of life and/or of death differently. Individual variation, in this sense, reflects personal upbringing and the uniqueness of one’s active construction of knowledge (79, 80).

The above mentioning is paramount to our discussion of the Life + Death Education Framework. Aside from formal documentations (e.g., a formal course outline for teaching purposes), it is poignant that we structure a positive social milieu (e.g., a positive learning environment) to help facilitate the embracement and acceptance of diversity. A conducive social milieu, in this analysis, may convey specific messages of acknowledgement of distinct personal historical backgrounds, epistemological beliefs, cultural values, etc. Moreover, such environmental-learning contexts may help a child to feel at ease with his or her own viewpoint, perspective, and/or cultural belief. The issue then, however, goes back to our original mentioning: whether it is possible for us to generalize the Life + Death Education to different learning-sociocultural settings. In other words, as a question for consideration, do we need to take into account the premise of universality when constructing and/or implementing the Life + Death Education Framework?

## 5 The ‘Life + Death Education Framework’: a brief overview

Our research collaborations, in terms of teaching and research development (e.g., our mentioning in Section 2 and Section 3), have led us to engage in several notable undertakings, for example: (i) conceptualizations of life education for empirical research studies (e.g., exploring the positive effect, *β*, of benevolent engagement on one’s state of emotional well-being), (ii) philosophical inquiries that seek to provide trans-mystical understanding of life and death experiences (e.g., does the notion of ‘post-death’ experience make logical sense?), (iii) conceptual-theoretical inquiries that relate the study of life and death education to other theoretical premises (e.g., the extent to which the study of positive psychology could relate to the teaching of life and death education), and (iv) curriculum development-related activities that may provide grounding and/or guidelines for quality teaching and learning purposes (e.g., development of the Life + Death Education Framework). These undertakings, we contend, are novel and creative and may, importantly, provide innovative sights for teaching and research advancement.

The Life + Death Education Framework, which we briefly mentioned earlier, was recently developed by us to help with quality teaching and curriculum development of life and (1–3) and death (4–6) education (81). We rationalize that, to date, there is no ‘universal’ blueprint or framework that we know of that may serve to assist educators with their curriculum structures. Even our own teaching of life and death education, for example, has involved the use of specific and purposive information (i.e., what we purposively choose to teach to students about life education). This lack of formalized information and/or proper structure has led us then, over the past several years, to consider developing a universal blueprint for guidance in terms of teaching and research purposes. This blueprint, or framework, consists of five interesting aspects of for educators, researchers, policymakers, etc. to consider:

i *Abroad subject overview that introduces the nature of life education and the nature of death education*:

That is, a detailed overview that describes the nature of both life and death education teaching.

ii *Specific learning outcomes (LOs) for student accomplishment*:

A designation of life education learning outcomes (e.g., Life Education LO-1: “To be able to understand the notion of ‘value contemplation’, which seeks to introduce the tenets of reflection, contemplation, and exploration of different core values, or life characteristics, for accomplishment.”) and death education learning outcomes (e.g., Death Education LO-1: “To learn and appreciate the transpersonal nature of death and dying.”) for students to accomplish (e.g., see Table 1 for full description).

**Table 1:** LOs for accomplishment.

LOs for accomplishment	Description
*Life Education LO-1*	*To be able to understand the notion of ‘value contemplation’, which seeks to introduce the tenets of reflection, contemplation, and exploration of different core life values and life characters for accomplishment.*
	This LO proposes the teaching of inner core life values (e.g., altruism) and life characters (e.g., trustworthiness), of a person – for example, his or her value of kindness to help others in society. Inner core values, we contend, form an important basis of a person’s holistic development.
*Life Education LO-2*	*To be able to understand and appreciate the fundamental issues of life functioning, including identification of various life trajectories and life courses that one may adopt and/or accomplish.*
	This LO proposes the teaching of different issues of life functioning, such as the ability for one to adapt to the contextual environment for effective functioning purposes. Relating to this is a focus on the meaning of what we term as ‘life courses’, or ‘life trajectories’. That a student should be able to recognize that every person has several or a number of life trajectories for accomplishment (e.g., a part-time student).
*Life Education LO-3*	*To explain how proactive life functioning may coincide with and/or explain the nature of human agency.*
	This LO proposes the teaching of human agency and its association or relationship with the teaching of life education. Proactive life functioning, an important aspect of life education, serves to support the understanding of human agency.
*Life Education LO-4*	*To recognize that the importance of the practice of ‘life cultivation’ or ‘cultivation of life qualities’ to help facilitate effective life functioning.*
	This LO proposes the teaching of the practice of cultivation for quality and/or effective life experiences. Cultivation is considered as a positive practice that may serve to facilitate, improve, and/or strengthen a person’s inner core life values or life characters (e.g., the strengthening of a person’s virtues).
*Death Education LO-1*	*To learn and appreciate the transpersonal nature of death and dying.*
	This LO uses death education teaching to introduce students to the concept of transpersonalism and the concept of mysticism or trans-mysticism. Students are encouraged to philosophize the nature of death and dying from a transpersonal point of view.
*Death Education LO-2*	*To identify different coping mechanisms or strategies that could help alleviate one’s fear and angst of death.*
	This LO considers different theoretical tenets and/or approaches (e.g., the study of trans-mysticism, which considers the importance of ‘self-transcendence’ experience) as coping mechanisms or strategies for usage.
*Death Education LO*-*3*	*To analyze and explain the intimate relationship between life and death.*
	This LO seeks to provide theoretical understanding that despite their unique characteristics or distinctive tenets, both life and death are interrelated with each other. That understanding of life functioning may, in turn, assist a person with his or her understanding of death.
*Death Education LO*-*4*	*To analyze and appreciate cross-cultural similarities and differences pertaining to understanding of death and dying.*
	This LO proposes the teaching of death and dying from a cross-cultural point of view. Cultural, ethnic, and racial groups differ in their perceptions, viewpoints, understandings, and approaches to the concept of death and dying (e.g., the ritual of ancestor worshipping). Accomplishing this LO may enable and/or assist students to appreciate the importance of anthropological grounding and historical-sociocultural context.

Note: Consult (81).

iii *Core competencies for student development*:

That is, a formal testament of expected ‘proficiencies’ or competencies (e.g., Core Competency 1: “To demonstrate capability to philosophize, self-reflect, and contemplate.”) pertaining to the learning of life and death education that educators want students to develop (e.g., see Table 2 for full description).

**Table 2:** Core competencies for consideration.

Core Competencies for accomplishment	Description
*Core Competency 1*	*To demonstrate capability to philosophize, self-reflect, and contemplate.*
	This core competency emphasizes a student’s capability to be able to philosophize, self-reflect, and/or contemplate about the nature of life and death. For example, a student’s philosophization may entail his/her belief, rationalization, and/or conviction that there is some form of existence beyond death itself.
*Core Competency 2*	*To demonstrate awareness of the contextual surrounding.*
	This core competency considers a student’s state of consciousness of his/her contextual surrounding (e.g., school environment) and its potential influences. Such competency may, in turn, enable and/or assist a student to act and behave accordingly.
*Core Competency 3*	*To demonstrate capability to think subjectively and objectively.*
	This core competency focuses on perception, judgment, rationalization, and reasoning of the broad life and death contexts at large. Moreover, this core competency informs and/or educates students about the uniqueness of the subjective perspective and the objective perspective of life and death.
*Core Competency 4*	*To be able to appreciate and accept non-conventional viewpoints, perspectives, ideas, opinions,* etc. *in a non-judgmental manner.*
	This core competency encourages students to be rationale, objective, and non-biased in his/her assessment, judgment, explanation, etc. of the world at large.
*Core Competency 5*	*To be able to recognize the importance of inner virtues* (e.g*., empathy*).
	This core competency encourages students to reflect or to introspect about their life values and characters – for example, what is so unique about me as an individual? It is poignant that students are able to identify life qualities (e.g., the ability to empathize) for cultivation purposes (i.e., the notion of ‘life cultivation’).
*Core Competency 6*	*To demonstrate creativity, innovation, and originality.*
	This core competency focuses on a student’s ability to be creative, innovative, and/or original in his/her thinking, perspective, etc. For example, a student’s innovation may consist of his/her proposition of a new meditation technique that may help to instill spirituality or spiritual faith. Likewise, a student’s creativity may involve his/her daily practice of Chinese calligraphy for therapy purposes.
*Core Competency 7*	*To recognize and appreciate the importance of one’s inner self.*
	This core competency, similar to Core Competency 5, focuses on introspective reflection about one’s own inner self. “Who am I as a person and what value(s) should I aspire to?” is an example of a student’s introspection. One notable distinction, in this case, is for a student to recognize his/her inner true self.
*Core Competency 8*	*To transform personal understanding into practice.*
	This core competency considers the importance of what is known as ‘active transformation’. Active transformation, where appropriate, is concerned with the application of theory into practice for positive or quality life experiences. For example, how could a student use his/her understanding of ‘Buddhist meditation’ to assist those in the local community?

Note: Consult (81).

**Figure 1:**
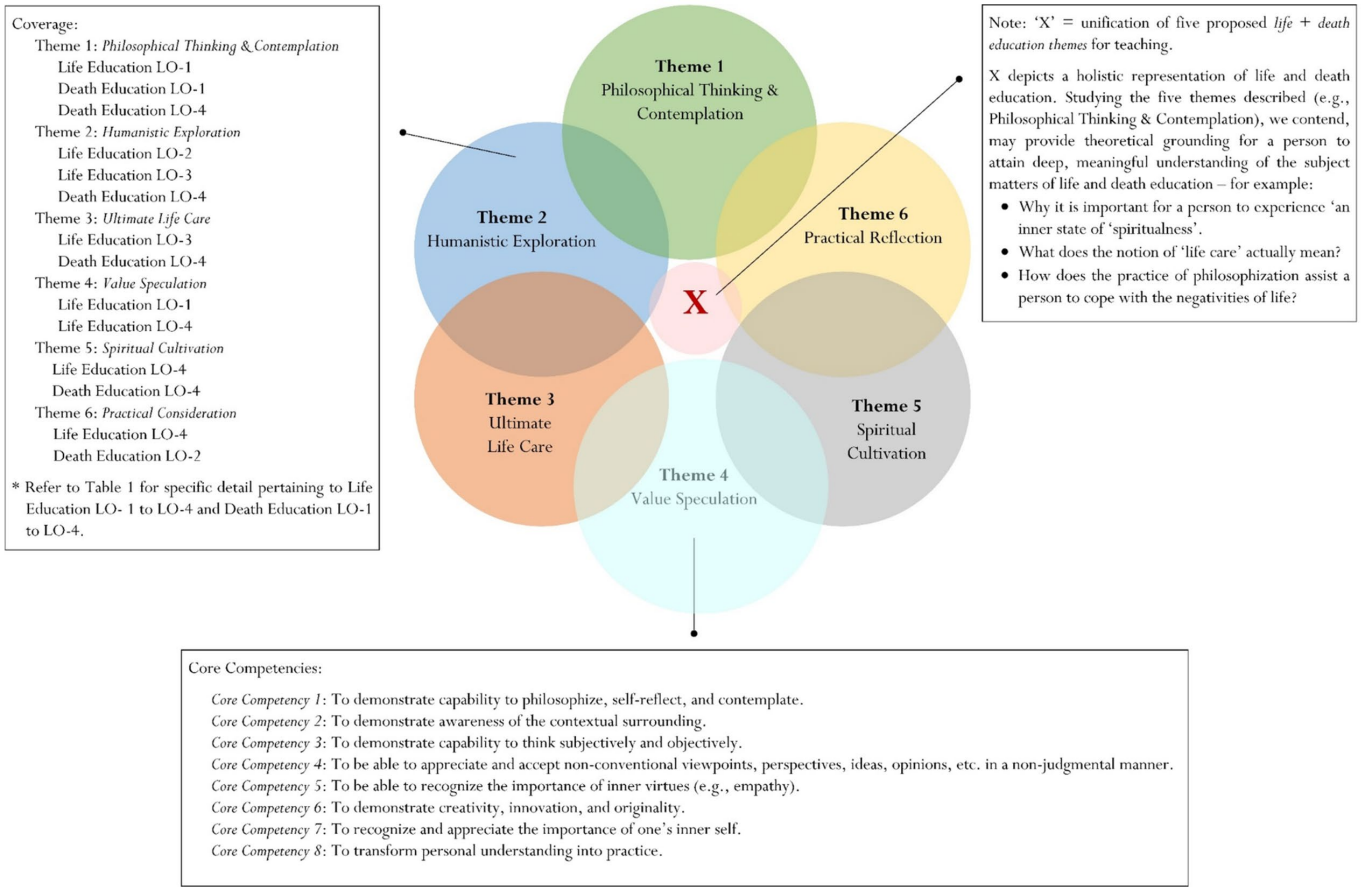
Universal framework of life and death education for consideration. Note: Consult (81).

iv *Specific learning themes for teaching and learning*:

Designated topical themes of life and death education, as shown in Figure 1 and Table 3 (see for full description), for coverage in terms of teaching and learning. Overall, there are six interrelated themes for consideration:

- *Philosophical Thinking & Contemplation*: is concerned with a person’s rational reflection of life knowledge, life skills, emotions, and attitudes that, in turn, may improve his or her ‘reflective thinking literacy’.
- *Humanistic Exploration*: reflects a person’s interest and intellectual curiosity to seek meaningful understanding into the nature of humanism – for example: “What is a human being?,” “Who am I as a person?,” “What is it like to be human?,” etc.
- *Ultimate Life Care*: relates to the issues of personal care, the true meaning of life, the intricate nature of suffering, grief, and suffering, and the aspiration, contemplation, and setting of personal goals for accomplishment.
- *Value Speculation*: seeks to explore a person’s subjective viewpoint, interpretation, reasoning, judgment, etc. of what values most in life.
- *Spiritual Cultivation*: is a positive endeavor that seeks to foster or cultivate one’s inner ‘incorporeal well-being’, which may consist of spiritual faith, spiritual wellness, spiritual understanding, and the like.
- *Practical Reflection*: emphasizes the process of ‘transformation’, which intimates the nexus between theory and applied practice (e.g., how can I utilize my knowledge of life and death to help others in the community?).

**Table 3:** Essential themes for consideration.

Themes for accomplishment	Description
Theme 1: *Philosophical Thinking and Contemplation*	‘Philosophical thinking’, in accordance with our conceptualization, is concerned with a person’s rational reflection of life knowledge, life skills, emotions, and attitudes that, in turn, may improve his or her ‘reflective thinking literacy’. Reflective thinking literacy relates to a student’s self-awareness, reasoned judgment, and understanding of biases, subjective interpretations, prejudices, etc. An ‘appropriate’ level of reflective thinking literacy, in this case, may enable a student to advance his or her interpersonal and/or intrapersonal development (e.g., social relationship or one’s ability to understand others). For example, a student’s self-awareness of a particular bias in-class (e.g., a teacher’s favoritism for a particular group of students) and her subsequent objective behavior may showcase her empathy, fairness, logical reasoning, etc.
Theme 2: *Humanistic Exploration*	‘Humanistic exploration’ is philosophical and somewhat transpersonal, reflecting a person’s interest and intellectual curiosity to seek meaningful understanding into the nature of humanism – for example: “What is a human being?”, “Who am I as a person?”, “What is it like to be human?”, etc. This emphasis is individual and subjective, reflecting one’s own unique interpretation, opinion, viewpoint, and understanding about the altruistic and esthetic nature of life. Engaging in humanistic exploration may enable and/or assist a person to introspect, contemplate, and acquire relevant life knowledge for theoretical understanding of human nature. That human nature, for example, suggests that as individuals, we are malleable to changes (e.g., contrasting viewpoints about sexuality) in accordance with the diverse contexts of time and space.
Theme 3: *Ultimate Life Care*	‘Ultimate life care’ relates to the issues of personal care, the true meaning of life, the intricate nature of suffering, grief, and suffering, and the aspiration, contemplation, and setting of personal goals for accomplishment. We contend that ultimate life care is an important theme that unifies life and death, life philosophy and religious faith, and epistemological beliefs and customary practices. The theme of ultimate life care, in this sense, serves to emphasize the importance of subjective well-being of a person. That, for example, we are mindful or self-aware of a student’s state of happiness, and/or his or her state of grief as he or she mourns for a loved one. Life fulfilment is not simply personal (e.g., a person’s attainment of financial wealth) but entails one’s ability to love and care for others in society.
Theme 4: *Value Speculation*	‘Value speculation’ seeks to explore a person’s subjective viewpoint, interpretation, reasoning, judgment, etc. of what values most in life. It is appropriate that in the age of technological advances that we appreciate the Internet, social media, and other related development. This theme, however, serves to educate and inform individuals of social, moral, and ethical issues that relate to the age of technological information. Moreover, value speculation also provides theoretical understanding to assist a person to appreciate and/or to recognize the importance of esthetic experiences in life. That life functioning does not simply consist of, entail, and/or conform to the operations of technological advances. One aspect of a person’s effective life functioning, in this case, relates to his or her self-awareness to judge, assess, and value the things that matter most in life.
Theme 5: *Spiritual Cultivation*	‘Spiritual cultivation’ is a positive endeavor that seeks to foster or cultivate one’s inner ‘incorporeal well-being’. This theme is abstract and philosophical for its emphasis on development of a person’s inner self, reflecting his or her state of self-actualization. Self-actualization, in this case, relates to a person’s pursuit of truth, love, self-transcendence, and ‘holiness’ or ‘divine connectedness’. Spiritual cultivation, in this sense, espouses a ‘spiritual’ dimension where one seeks to know more about the meaning of eternity or infinity (e.g., is there life beyond death or can one’s soul exist and/or transverse infinitely?). We contend that this topical theme is transpersonal and somewhat abstract, reflecting an important focus on what we term as ‘an inner state of ‘spiritualness’. Cultivation of ‘inner spiritualness’, from our viewpoint, may assist a person to experience life differently.
Theme 6: *Practical Reflection*	‘Practical reflection’, in accordance with our conceptualization, emphasizes the nexus between theory and applied practice. That theoretical understanding of life and death education has a practical focus for consideration. This theme recognizes the importance of the tenet of ‘transformation’ – that is, how can we transform what we know into practice? For example, in relation to death, consider the issue of grief and suffering and how one could use theoretical understanding of ‘death education’ to help placate such negativity. In a similar vein, in relation to proactive life functioning, how would a teenager use his or her understanding of mindfulness and meditation to help him or her with the final exams? Unlike the other five themes for learning and accomplishment (e.g., Theme 1: Philosophical Thinking and Contemplation), this theme is non-philosophical in makeup.

Note: Consult (81).

v *Specific elements*:

Designated elements, which there are four, for learning, attainment, and appreciation (e.g., Element: ‘Application’, which emphasizes the promotion of the application of the life and death education premise) (e.g., see Table 4 for full description).

**Table 4:** Elements of the Life + Death Education Framework.

Elements	Description
i *Application*	The promotion of the application of the life and death education (2, 4, 8, 36) premise may serve to facilitate and/or to enhance a person’s subjective well-being, which consists of different life values (e.g., gratitude) and life characters (e.g., respect) or life qualities (e.g., person’s empathetic understanding and/or his or her kindness). For example, application of deep, meaningful understanding of spirituality (32, 98, 99) may consist of a person’s ‘higher-order’ meditative practice, which in turn could help her experience a state of self-transcendence (100, 101).
ii *Growth*	To promote the life and death education premise as an alternative theoretical standing (e.g., in tandem with or as opposed to, say, the theoretical premise of positive psychology (102, 103) that may be used to promote individual growth) for usage to help encourage, promote, and/or facilitate a person’s individual growth (e.g., his or her personal experience of flourishing). For example, engaging in ultimate life care (i.e., Theme 3) and/or spiritual cultivation (Theme 5), where appropriate, may help a person to attain an internal state of self-fulfilment, which is positive and self-gratifying.
iii *Remedy and Appreciation*	Life and death education (2, 4, 8, 36), via means of deep, meaningful understanding and/or application of its tenets into practice, may function as a remedy and, in the process, help to instill appreciation for the intricate nature of life and death. Life in itself is complex with uncertainties, obstacles, mishaps, etc. Feeling somewhat down or negative about life, at any moment in time, requires some form of remedy or remediation that may, in this case, involve the use of life and death education. Humanistic exploration about the intricacies of life itself (Theme 2) may, in this sense, offer some rewarding appreciation about one’s own luck or fortunate circumstances (e.g., that one is fortunate to not having to live in poverty).
iv *Humanistic Understanding + Life Enlightenment*	Appreciating the importance of life and death education (2, 4, 8, 36), in part, reflects and/or relates to a person’s ‘humanistic understanding’ about himself or herself. Humanistic understanding entails exploration, introspection, and enlightenment of one’s inner self (e.g., a student’s seeking to understand the impact of his quality life characteristics) in order to live a cherished and meaningful life. We rationalize that humanistic understanding is a positive and life-fulfilling endeavor that offers critical insights for growth purposes. For example, upon introspection, a student may identify 1–2 distinct life characteristics for cultivation. Cultivation of life characteristics such as say kindness, generosity, and/or empathy is meaningful and self-actualized.

Note: Consult (81).

**Figure 2:**
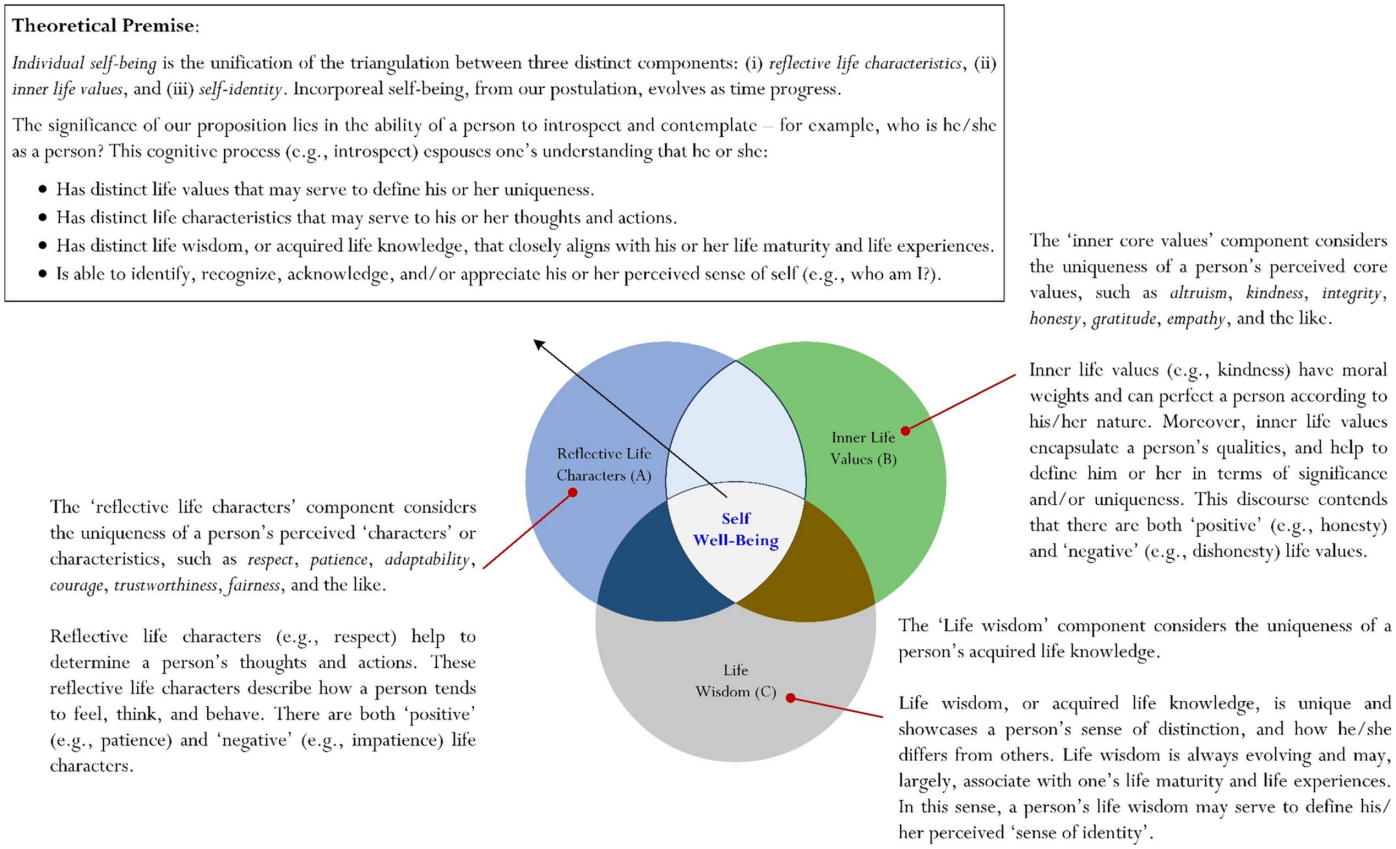
Conceptualization of ‘self well-being’.

Overall, then, the brief overview of the Life + Death Education Framework above showcases the important structures and/or guidelines for adherence to when one teaches the subject of life and death (3, 10, 17, 18). Moreover, we contend that the uniqueness of the Life + Death Education Framework lies in its established grounding that may enable and/or assist researchers with advancement and development of new theoretical premises, associations, concepts, etc. (e.g., the extent to which *positive psychological teaching* could help alleviate the perceived feeling of grief) (3). In this analysis, we purport that it is plausible to use the Life + Death Education Framework (i.e., the implementation of the Life + Death Education Framework) as theoretical grounding to conceptualize an alternative nomenclature and/or concept, which we term as ‘self well-being’. In the next section of the article, we explore in detail our conceptualization (e.g., Figure 2): *that the Life + Death Education Framework may establish theoretical grounding to support the underlying nature of the proposed concept known as self well-being*.

## 6 Introducing the concept of ‘self well-being’

One important outcome of the school system or the schooling processes entails the fostering of positive well-being experiences (82, 83). This noting contends that successful schooling is more than just one’s ability to achieve exceptional school grades. In this analysis, successful schooling may involve a wide range of school-based experiences, such as one’s testimonial evidence of his or her positive well-being (16, 84). Moreover, as the literature (15, 83, 85–88) attests, positive well-being may serve as a dynamic ‘driver’ of change, helping to direct and govern a student’s behaviors, thought patterns, feelings for others, etc.

A prevalent line of inquiry exists at present, namely, what is personal well-being? This research question seeks to attain relevant insights into the *meaning* and *underlying nature* of personal well-being. Similar to the concept of *intelligence* (89–92), it is somewhat difficult for us to derive and/or to provide a clear, consistent definition of personal well-being. In a documentation published some years back, for example, Fraillon (15) provided a comprehensive overview of personal well-being with reference to the school contexts (i.e., the study of ‘student well-being’). According to the author’s summary, personal well-being (i.e., in this case, student well-being) is a ‘multifaceted construct’ that consists of different domains or components – for example: *physical* well-being, *economic* well-being, *psychological* well-being, *cognitive* well-being, and *social* well-being (93). Overviews from the Australian Catholic University (85), Dodge et al. (14), Pollard and Lee (93), Soutter (16), and others, likewise, serve to illustrate the complexity of the nature of personal well-being.

Our analysis of the literature has led to our identification of a number of key words and/or phrases that may depict the operational functioning of personal well-being – for example:

- *Functioning*: A person’s (e.g., student) internal state of functioning on a daily basis, including emotional, social, cognitive, and spiritual.
- *Adaptation*: A person’s (e.g., student) ability to successfully adapt to his or her contextual environment (e.g., classroom).
- *Multidimensionality*: That personal well-being (e.g., student well-being) is multidimensional or multifaceted, consisting of different domains, elements, factors, etc.
- *Self-fulfilment of Needs*: There is an inherent need for a person (e.g., student) to self-fulfil his or her inner needs (e.g., psychological needs).
- *Positivity*: That personal well-being (e.g., student well-being) is a positive construct or that it entails positive experiences. Having said this, however, it may be argued that there is also a negative scope (e.g., negative well-being experience).
- *Optimal Experience*: That a person’s well-being (e.g., student well-being) reflects his or her optimal experiences (e.g., optimal social experience).
- *Personal Satisfaction*: That personal well-being (e.g., student well-being), positive in experience, reflects and/or showcases one’s internal state of satisfaction.

Our considered interpretation of personal well-being, termed as self well-being or ‘individual self-being’ (Figure 2), is somewhat unique for its humanistic and ‘life functioning’ grounding. In this analysis, our interpretation of personal well-being uses the theoretical lens of life education teaching (e.g., our use of the Life + Death Education Framework), which differ from the existing psychological perspective (15, 85). Innovatively, the life education lens places emphasis on the practice of ‘introspection’ or introspective reflection (e.g., Theme 1: Philosophical Thinking and Contemplation, Theme 2: Humanistic Exploration), which is subjective and somewhat transpersonal. Using Theme 1 from the Life + Death Education Framework as theoretical grounding, we define introspection as:

*A cognitive process, which directs a person to reflect on internalized information from the contextual environment for the purpose of seeking awareness, understanding, and appreciation of his or her inner self.*

The underlying discourse then, in accordance with our proposition, is that engagement of introspection is paramount to the formation of self well-being. In this analysis, we theorize that one’s self-awareness of personal well-being experiences involves active introspective reflection, which is individual, measured, and contemplative. As such, the ‘evolution’ of a person’s self well-being is continuous and intimately associates with the process of introspection.

### 6.1 The underlying nature of self well-being

As shown in Figure 2, the nature or the formation of self well-being, or individual self-being, is made up of the unification of three distinct components: ‘reflective life characters’ (e.g., a person’s ‘trustworthiness’), ‘inner life values’ (e.g., a person’s ‘altruistic state’), and ‘life wisdom’ (e.g., a person’s acquired knowledge about the conflicting emotions that arise from personal experience of poverty)(See Figure 2 for detail). Central to this conceptualization is the theorization that one’s practice of introspection or introspective reflection, as defined earlier, may serve to facilitate the formation of self well-being. Every person’s self well-being, in this analysis, is unique and differs from another person’s self well-being.

Our conceptualization, derived from life education teaching (e.g., the use of the Life + Death Education Framework) (3, 10, 17, 18), contends that the underlying nature of self well-being (e.g., what is one’s personal well-being experience?) is more humanistic and transpersonal, involving a person’s inner thoughts, introspection, and philosophization. In this sense, the nature of self well-being is philosophical and open-ended, reflecting one’s attempts to introspect in order to obtain deep, meaningful understanding of the ‘inner self’, which is unique and serves to define one’s perceived sense of self-identity. Self-identity, a unification of life characters, inner life values, and life wisdom, we contend, is the embodiment of one’s ‘humanistic beingness’. That a person’s self-identity is unique and serves to define and/or espouse his or her well-being experiences. This theoretical premise, from our point of view, is significant as it:

- Distinguishes our unique interpretation of personal well-being, which is humanistic and transpersonal, from other existing theoretical positions (e.g., the psychological position of personal well-being, which emphasizes the importance of both interpersonal and intrapersonal experiences) (15, 85). This interpretation suggests then that personal well-being, or humanistic beingness, is largely a construction of one’s own interpretation of oneself (e.g., what is an important life character or inner life value that I see as being most profound….?).
- Seeks to accentuate, in part, the importance of the concept of ‘self’ (8, 94–96), which delves into a person’s understanding of his or her inner nature (e.g., what is it that represents me?). Our theorization, again differing from existing theoretical viewpoints (e.g., psychological viewpoint), is unique for its attempt to relate the nature of personal well-being with the nature of self – hence, our coining of the term ‘self well-being’ (i.e., self + well-being).
- Emphasizes the uniqueness of acquired life wisdom (33–35), or life knowledge, as an entity of personal well-being. This conceptualization is indeed unique for its rationalization: that one’s personal well-being at the present time indicates, in part, a certain level of life wisdom, or that, alternatively, one’s acquired life wisdom level serves to highlight one’s personal well-being.

Overall, then, we contend that our use of the Life + Death Education Framework to offer an alternative interpretation of personal well-being is novel and innovative. Central to this thesis is the rationalization that personal well-being is intimately linked to the study of life and death education (3, 6, 9, 10). This considered viewpoint contends that a person’s perceived life functioning on a daily basis may serve to define his or her well-being. Perceived life functioning, in this analysis, involves a person’s introspective reflection of his or her inner self. The notion of ‘self-identity’, a contextual term that we have coined, plays an integral part in our alternative theorization of personal well-being. Self-identity, as conceptualized in Figure 2, is a ‘façade’ or a ‘self-image’ of a person that is unique, depicting his or her reflective life characters, inner life values, and acquired life wisdom. Every person’s self-identity is distinguished, showcasing his or her ‘deep, inner core’.

## 7 Final thought and caveats for consideration

The Life + Death Education Framework, as overviewed in the preceding sections, is still in its early stage of evolution in terms of usage, implementation, research undertaking, etc. We acknowledge the potential limitations or caveats that may give rise to further advancement and development – for example, in terms of universality, can we apply or implement the Life + Death Education Framework to different learning-sociocultural contexts? By all accounts, our sample example regarding the use of the Life + Death Education Framework to explain the underlying nature of personal well-being (i.e., development of the concept ‘self well-being’) is simply speculative. Theoretical-conceptual inquiries (e.g., our proposition of the Life + Death Education Framework), using philosophical psychology (8, 30, 97) as a logical basis require robust empirical validation.

We urge educators, researchers, policymakers, etc. to use our Life + Death Education Framework for teaching and/or research purposes. There are several recommendations that are noteworthy for consideration. Firstly, consider refining the Life + Death Education Framework (e.g., refining the Life Education LOs) for the main purpose of contextualization (e.g., the Taiwanese learning-sociocultural context), relevance, and suitability (e.g., can a modified version of the Life + Death Education Framework be used to teach Japanese university students?). Secondly, consider using the Life + Death Education Framework as theoretical grounding for the conceptualization of a new theoretical premise (e.g., can our original version or a modified version of the Life + Death Education Framework be used to advance theoretical understanding of Public Palliative Care Education?). Thirdly, in terms of teaching and learning experiences, consider the potential generalization and/or the ‘universality’ of the Life + Death Education Framework (e.g., does the Life + Death Education Framework have the same meaning for different learning-sociocultural contexts?).

## 8 Conclusion

The study of life and death education (3, 6, 9, 10) is unique for its pragmatic, philosophical, and humanistic nature. Unlike other academic subjects and fields of research (e.g., Calculus), life and death education teaching may consist of subjective, non-definitive, and non-conclusive interpretations and understandings (e.g., what is the true meaning of life?). It is somewhat difficult, from our point of view, to streamline and/or to show consistency when teaching life and death education. For example, is it valid or logical for us to consider the premise of saṃsāra (64, 65), and/or the concept of ‘reincarnation’ (66–68) when teaching life and death education? Our teaching and research development over the past decade has led us to undertake an interesting discourse: the proposition of a blueprint or framework known as the ‘Life + Death Education Framework’.

The Life + Death Education Framework is unique for its theoretical tenets (e.g., LOs for accomplishment, Core Competencies for development), serving as guidelines and structures for teaching and research purposes. For example, in terms of teaching and learning, we purport that the Life + Death Education Framework may provide theoretical grounding to assist educators in their curriculum development of life and death education (e.g., what is a relevant theme pertaining to death education that is noteworthy for inclusion?). In a similar vein, for research purposes, we contend that the Life + Death Education Framework may offer informative insights, which could help researchers to formulate new ideas, theoretical premises, etc. In this analysis, as overviewed in the latter sections of this article, we used the Life + Death Education Framework to frame our conceptualization of concept, which we termed as ‘self well-being’.

## Data Availability

The original contributions presented in the study are included in the article/supplementary material, further inquiries can be directed to the corresponding author.

## Footnotes

^1^Source: https://newsinhealth.nih.gov/2017/10/coping-grief, https://www.unicef.org/armenia/en/stories/strategies-cope-grief, https://www.apa.org/topics/families/grief.

^2^Source: https://www.ekrfoundation.org/elisabeth-kubler-ross/, https://hdsa.org/wp-content/uploads/2015/02/13080.pdf.

^3^Source: https://byjus.com/maths/algebraic-equations/.
